# Identification of Metabolic Biomarkers in Relation to Methotrexate Response in Early Rheumatoid Arthritis

**DOI:** 10.3390/jpm10040271

**Published:** 2020-12-10

**Authors:** Helen R. Gosselt, Ittai B. Muller, Gerrit Jansen, Michel van Weeghel, Frédéric M. Vaz, Johanna M. W. Hazes, Sandra G. Heil, Robert de Jonge

**Affiliations:** 1 Amsterdam Gastroenterology and Metabolism, Department of Clinical Chemistry, Amsterdam UMC, VUmc, 1081 HV Amsterdam, The Netherlands; i.muller@amsterdamumc.nl (I.B.M.); r.dejonge1@amsterdamumc.nl (R.d.J.); 2 Department of Clinical Chemistry, Erasmus MC University Medical Center Rotterdam, 3015 GD Rotterdam, The Netherlands; s.heil@erasmusmc.nl; 3 Amsterdam Rheumatology and Immunology Center, Amsterdam UMC, VUmc, 1081 HV Amsterdam, The Netherlands; g.jansen@amsterdamumc.nl; 4 Amsterdam Gastroenterology Endocrinology Metabolism, Laboratory Genetic Metabolic Diseases, Departments of Clinical Chemistry and Pediatrics, Amsterdam UMC, University of Amsterdam, 1105 AZ Amsterdam, The Netherlands; m.vanweeghel@amsterdamumc.nl (M.v.W.); f.m.vaz@amsterdamumc.nl (F.M.V.); 5 Core Facility Metabolomics, Amsterdam UMC, University of Amsterdam, 1105 AZ Amsterdam, The Netherlands; 6 Department of Rheumatology, Erasmus MC University Medical Center Rotterdam, 3015 GD Rotterdam, The Netherlands; j.hazes@erasmusmc.nl; 7 Academic Center of Excellence−Inflammunity, Erasmus MC University Medical Center, 3015 GD Rotterdam, The Netherlands

**Keywords:** methotrexate, rheumatoid arthritis, metabolomics, biomarkers, treatment response

## Abstract

This study aimed to identify baseline metabolic biomarkers for response to methotrexate (MTX) therapy in rheumatoid arthritis (RA) using an untargeted method. In total, 82 baseline plasma samples (41 insufficient responders and 41 sufficient responders to MTX) were selected from the Treatment in the Rotterdam Early Arthritis Cohort (tREACH, trial number: ISRCTN26791028) based on patients’ EULAR response at 3 months. Metabolites were assessed using high-performance liquid chromatography-quadrupole time of flight mass spectrometry. Differences in metabolite concentrations between insufficient and sufficient responders were assessed using partial least square regression discriminant analysis (PLS-DA) and Welch’s *t*-test. The predictive performance of the most significant findings was assessed in a receiver operating characteristic plot with area under the curve (AUC), sensitivity and specificity. Finally, overrepresentation analysis was performed to assess if the best discriminating metabolites were enriched in specific metabolic events. Baseline concentrations of homocystine, taurine, adenosine triphosphate, guanosine diphosphate and uric acid were significantly lower in plasma of insufficient responders versus sufficient responders, while glycolytic intermediates 1,3-/2,3-diphosphoglyceric acid, glycerol-3-phosphate and phosphoenolpyruvate were significantly higher in insufficient responders. Homocystine, glycerol-3-phosphate and 1,3-/2,3-diphosphoglyceric acid were independent predictors and together showed a high AUC of 0.81 (95% CI: 0.72–0.91) for the prediction of insufficient response, with corresponding sensitivity of 0.78 and specificity of 0.76. The Warburg effect, glycolysis and amino acid metabolism were identified as underlying metabolic events playing a role in clinical response to MTX in early RA. New metabolites and potential underlying metabolic events correlating with MTX response in early RA were identified, which warrant validation in external cohorts.

## 1. Introduction

Rheumatoid arthritis (RA) is a chronic autoimmune disease affecting joint linings, resulting in pain and inflammation [1]. Methotrexate (MTX) is the first-line therapy in rheumatoid arthritis (RA); however, treatment strategies still consist of trial and error [2]. MTX is an antifolate with a long background in cancer chemotherapy acting as a potent inhibitor of folate metabolism impacting numerous targets in one-carbon metabolism, nucleotide and amino acid biosynthesis [3]. The mechanism of MTX in RA is still not fully understood, which is why it is still unknown why some patients respond better than others to MTX [4,5]. Response to MTX-based therapy can be determined after 3 to 6 months according to changes in disease activity score 28 (DAS28) and insufficient responders require step-up treatment with biologic disease modifying anti rheumatic drugs (bDMARDs; e.g., TNF-alpha inhibitors, IL-6 inhibitors [6]) or targeted synthetic DMARDs (tsDMARDs, e.g., Janus-kinase inhibitors [7,8]) as described in the EULAR recommendations for the management of RA [2]. To enable quicker treatment adjustments, earlier identification of insufficient responders to MTX will be of great clinical importance in personalized medicine.

Several studies investigated baseline biomarkers to predict clinical response to MTX at 3 and/or 6 months in a targeted way [9]. We have previously developed [10] and externally validated [11] a baseline clinical prediction model for insufficient response to MTX. Apart from clinical predictors, this prediction model includes biomarkers such as erythrocyte folate and adenosine triphosphate (ATP) binding cassette (ABC) transporter polymorphisms. Applying an untargeted approach might reveal new and overlooked biomarkers and provide new insights into the etiology of non-response to MTX. Others have shown that RA patients have a different serum metabolite signature compared to healthy controls [12,13,14,15]. Study results from a literature review showed that essential amino acids (citric acid, isoleucine, methionine, valine) and non-essential amino acids (threonine, histidine and alanine) were consistently lower in RA patients compared to healthy controls [16]. Additionally, differences in metabolic profiles have been associated with different stages of disease [14,15] as well as in relation to treatment response [17,18,19,20]. The aim of the current study was to identify potential baseline biomarkers in treatment-naive patients for the prediction of insufficient response to MTX at 3 months in RA patients using an untargeted approach.

## 2. Materials and Methods

### 2.1. Materials and Subjects

Baseline plasma samples of 82 early RA patients were selected from the treatment in the Rotterdam early arthritis cohort (tREACH; ISRCTN registered trial, number: ISRCTN26791028) [21], based on plasma availability and their European League Against Rheumatism (EULAR) response at 3 months, including 41 insufficient responders and 41 sufficient responders. Insufficient response was defined as: 3-month DAS28-ESR > 5.1 and improvement of DAS28-ESR ≤ 1.2. Sufficient response was defined as: 3-month DAS28-ESR ≤ 3.2 and improvement in DAS28-ESR > 1.2 over the first 3 months. All subjects received MTX (combination) therapy (see Table 1) and all accomplished the American College Rheumatism (ACR)/EULAR 2010 classification criteria for rheumatoid arthritis (RA) [22].

After blood collection in ethylenediamine tetraacetic acid (EDTA) tubes, samples were immediately placed on ice, followed by centrifugation for 10 min at 1700× *g* at a temperature of 4 °C. Plasma samples were stored at −80 °C, as previously described [23]. This study was approved by the medical ethics committee of Erasmus Medical Center (MEC-2006-252) and written informed consent was obtained for included patients. All procedures performed were in accordance with the 1964 Helsinki Declaration and its later amendments.

### 2.2. Metabolomics Study

Metabolomics analysis was performed using a semi-quantitative analysis at the Core Facility Metabolomics of the Amsterdam UMC as described previously [24]. In short, a mixture of 75 µL of the following internal standards in water was added to 25 µL plasma: adenosine-^15^N_5_-monophosphate (100 µM), adenosine-^15^N_5_-triphosphate (100 µM), D_4_-alanine (100 µM), D_7_-arginine (100 µM), D_3_-aspartic acid (100 µM), D_4_-citric acid (100 µM), ^13^C_1_-citrulline (100 µM), ^13^C_6_-fructose-1,6-diphosphate (100 µM), guanosine-^15^N_5_-monophosphate (100 µM), guanosine-^15^N_5_-triphosphate (100 µM), ^13^C_6_-glucose (1 mM), ^13^C_6_-glucose-6-phosphate (100 µM), D_3_-glutamic acid (100 µM), D_5_-glutamine (100 µM), ^13^C_6_-isoleucine (100 µM), D_3_-leucine (100 µM), D_4_-lysine (100 µM), D_3_-methionine (100 µM), D_6_-ornithine (100 µM), D_5_-phenylalanine (100 µM), D_7_-proline (100 µM), ^13^C_3_-pyruvate (100 µM), D_3_-serine (100 µM), D_5_-tryptophan (100 µM), D_4_-tyrosine (100 µM), D_8_-valine (100 µM). Subsequently, 425 µL water, 500 µL methanol and 1 mL chloroform were also added and the samples were mixed and centrifuged for 10 min at 14,000 rpm. The polar phase was dried using a vacuum concentrator at 60 °C. Subsequently, dried samples were reconstituted in 100 µL methanol/water (6/4; *v/v*). Then, 5 µL metabolic extract was injected onto a SeQuant 100 × 2.1 mm ZIC-cHILIC column, 3 μm particle diameter (Merck, Darmstadt, Germany). The column temperature was maintained at 30 °C and samples at 12 °C during analysis. An impact II quadrupole time of flight (QTOF) (Bruker Daltoniks) mass spectrometer (MS) was used in the negative and/or positive electrospray ionization mode where mass spectra of the metabolites were obtained by continuous scanning from *m*/*z* 50 to *m*/*z* 1200 with a resolution of 50,000 full half-maximum width (FHMW). Data were analyzed using Bruker TASQ software version 2.1.22.3. All reported metabolite intensities were normalized to internal standards with comparable retention times and response in the MS. Metabolite identification was based on a combination of accurate mass, (relative) retention times and fragmentation spectra, compared to the analysis of a library of standards. Statistical analysis and visualization of the acquired data were done in a R environment using the ggplot2, ropls and mixOmics packages [25,26,27]. Identified metabolites were classified according to the Human Metabolome Database [28].

### 2.3. Statistics

Mean and standard deviation (± SD) between baseline group characteristics were compared using a two-sample *t*-test. Proportions in baseline characteristics were compared using a two-proportion test in R. To identify metabolites that could discriminate insufficient responders from sufficient responders, we used partial least square regression discriminant analysis (PLS-DA). Variable Importance Projection (VIP) scores were examined to select best discriminating variables, where a VIP score of ≥1 was considered important [29]. Furthermore, to investigate differences in mean concentrations between response groups at baseline, a Welch’s *t* test was performed and fold changes were calculated, which were together visualized in a volcano plot. We corrected for multiple comparisons using the Benjamini–Hochberg method. A multivariable model was built with metabolites that were significantly different between insufficient and sufficient responders and had a VIP score >1. As highly correlated variables could influence logistic regression, correlations between metabolites were first assessed using Pearson’s correlation in a correlation matrix using the “corrplot” package in R. In the same analysis, the relation between metabolites and inflammatory factors (erythrocyte sedimentation rate [ESR] and C-reactive protein [CRP]) was assessed to examine whether the metabolites were a surrogate for inflammation. Metabolites with a Pearson’s correlation coefficient of >0.6 were considered strongly correlated. In case two metabolites were strongly correlated, only the metabolite with the highest VIP score in relation to response was included in the model. From the model, a receiver operating characteristic (ROC) curve with area under the curve (AUC) was produced. Sensitivity and specificity were calculated using the “pROC” package in R. In addition, non-linear relationships between metabolites and the outcome were examined in a random forest analysis, which is an ensemble classification method. For the random forest analysis, a random seed was set to 415 to make the analysis reproducible. Mean decrease in accuracy (how well the model performs) and decrease in Gini score (how pure the nodes are at the end of the tree) were assessed to evaluate variable importance upon removal of each variable. Hence, the larger the decrease in accuracy and Gini score, the more important the variable.

To obtain a better understanding of which metabolic pathways were enriched between insufficient and sufficient responders to MTX, an overrepresentation analysis (ORA) was performed using the online “Metabolite Set Enrichment Analysis” (MSEA) tool as integrated in the MetaboAnalyst software 4.0 [30]. Compound names of metabolites with a VIP score >1 produced by the PLS-DA analysis were used as input. Small molecule pathway database (SMPDB) was selected as reference library containing 99 metabolite sets based on normal human metabolic pathways. A hypergeometric test was performed to evaluate if combinations of differentially expressed metabolites were represented more than expected by chance, providing a one-tailed *p*-value. *p*-values were adjusted for multiple testing using the Holm–Bonferroni method and false discovery rate (FDR) according to the Benjamini–Hochberg method.

## 3. Results

### 3.1. Baseline Comparisons

Mean baseline DAS28 was lower in RA patients with insufficient response to MTX therapy (4.3 ± 1.3) compared to RA patients with sufficient response to therapy (5.6 ± 1.0, *p* < 0.001; Table 1), while BMI was higher in the insufficient responder group (*p* < 0.001; Table 1). Other characteristics such as age, sex, rheumatoid factor (RF) positivity, anti-citrullinated protein antibody (ACPA) positivity and medication were similar between both groups.

### 3.2. Metabolite Analysis

**Figure 1:**
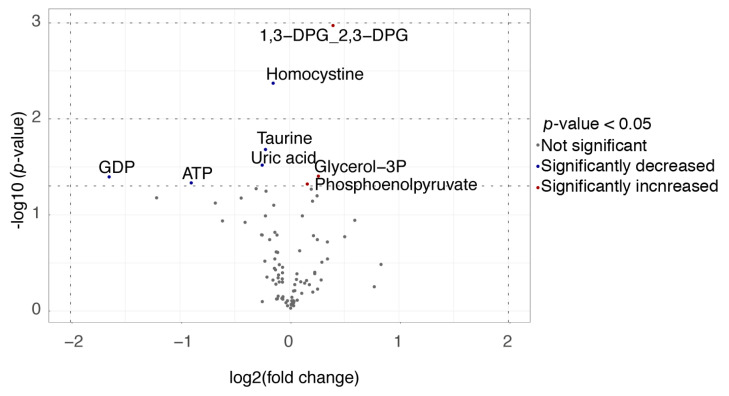
Volcano plot of significantly different metabolites in insufficient responders (DAS28-ESR > 3.2) and sufficient responders (DAS28-ESR ≤ 3.2).

Metabolites were examined as a potential biomarker for response to MTX. A list of the 50 most important variables was created according to their VIP scores from to the PLS-DA analysis (Supplementary Figure S1) and *p*-values acquired from Welch’s *t*-test, which is presented in Supplementary Table S1. Moreover, 1,3-diphosphoglyceric acid (DPG)/2,3-DPG and homocystine had the largest VIP scores (2.439 and 1.927, respectively) and were most significantly different between insufficient responders and sufficient responders (*p* = 0.001 and *p* = 0.004, respectively; Table S1). Homocystine, taurine, adenosine triphosphate (ATP), guanosine diphosphate (GDP) and uric acid concentrations were significantly lower in insufficient responders versus sufficient responders, while 1,3-diphosphoglyceric acid (1,3-DPG) and 2,3-diphosphoglyceric acid (2,3-DPG), glycerol-3-phosphate and phosphoenolpyruvate (PEP) were significantly higher in insufficient responders versus sufficient responders (Table S1 and Figure 1).

From the significantly different metabolites, GDP had the largest log2 fold change (1.647) as depicted in a volcano plot (Figure 1). No significant differences were observed after correction for multiple testing.

**Figure 2:**
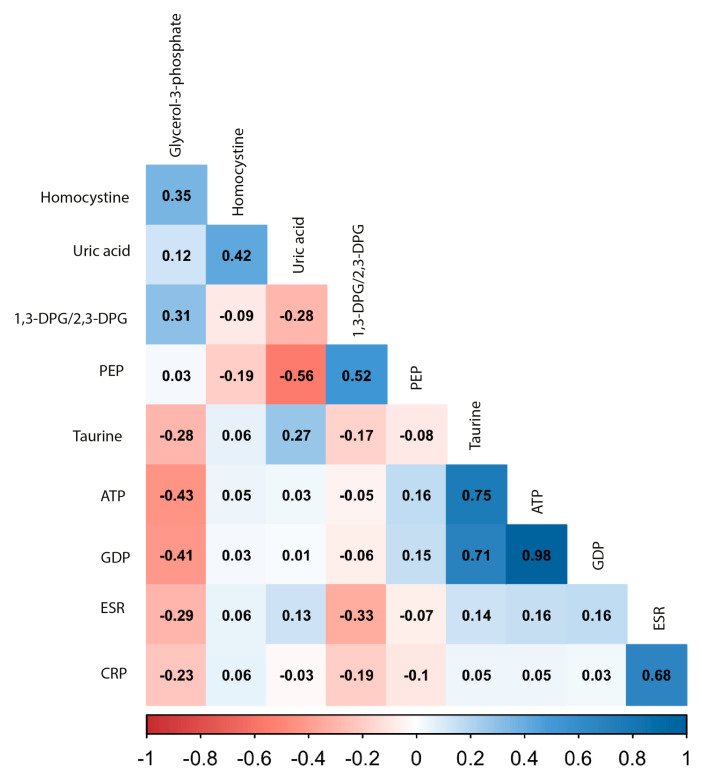
Correlation matrix between significantly different metabolites at baseline and inflammatory factors. Included metabolites shown were significantly different in relation to response at 3 months according to results of a Welch’s *t*-test. The color indicates the strength of the correlation: dark red indicates a strong negative correlation and dark blue a strong positive correlation. The Pearson’s correlation coefficient is printed in the squares. Erythrocyte-sedimentation rate (ESR) and C-reactive protein (CRP) were added as a proxy for inflammation.

It should be noted that, in this study, we aimed to find a new biomarker for response and not another surrogate marker for inflammation, such as the erythrocyte-sedimentation rate (ESR) or C-reactive protein (CRP). To examine whether the most promising candidate metabolites were independent of inflammation, we examined their correlation with ESR and CRP (Figure 2).

**Figure 3:**
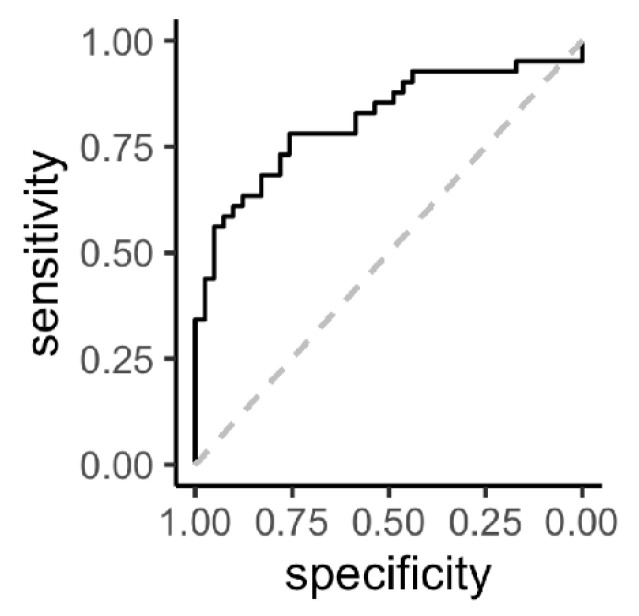
Receiver operating characteristic (ROC) curve (black solid line) of prediction of insufficient response (DAS28-ESR > 3.2) including significantly different metabolites in relation to response at 3 months. Predictors included in the model were: baseline homocystine, glycerol-3-phosphate and 1,3-diphosphoglyceric acid/2,3-diphosphoglyceric acid. The grey dotted line represents “the line of no discrimination”.

All correlations with ESR and CRP were weak (Pearson’s correlation coefficient r < 0.33), indicating that these metabolites do not reflect inflammation. The most significant metabolites were analyzed together in a multivariable logistic regression model to assess their performance as biomarkers in predicting insufficient response to MTX, including: homocystine, PEP, glycerol-3-phosphate, 1,3-DPG/2,3-DPG, uric acid and taurine. ATP and GDP concentrations were also significantly different between response groups; however, these were highly correlated with taurine (Figure 2). Of this model, a receiver-operating characteristic (ROC) plot was constructed with an area under the curve (AUC) of 0.82 (95% CI: 0.73–0.91). From all predictors in the model, only homocystine (*p* = 0.007) and glycerol-3-phosphate (*p* = 0.020) were significant independent predictors, while 1,3-/2,3-DPG was borderline significant (*p* = 0.080), for which reason we reduced the model to these three predictors. Using the combination of these predictors, a new ROC curve was constructed with an AUC of 0.81 (95% CI: 0.72–0.91; Figure 3) and corresponding sensitivity of 0.78 and specificity of 0.76.

### 3.3. Random Forest Analysis

**Figure 4:**
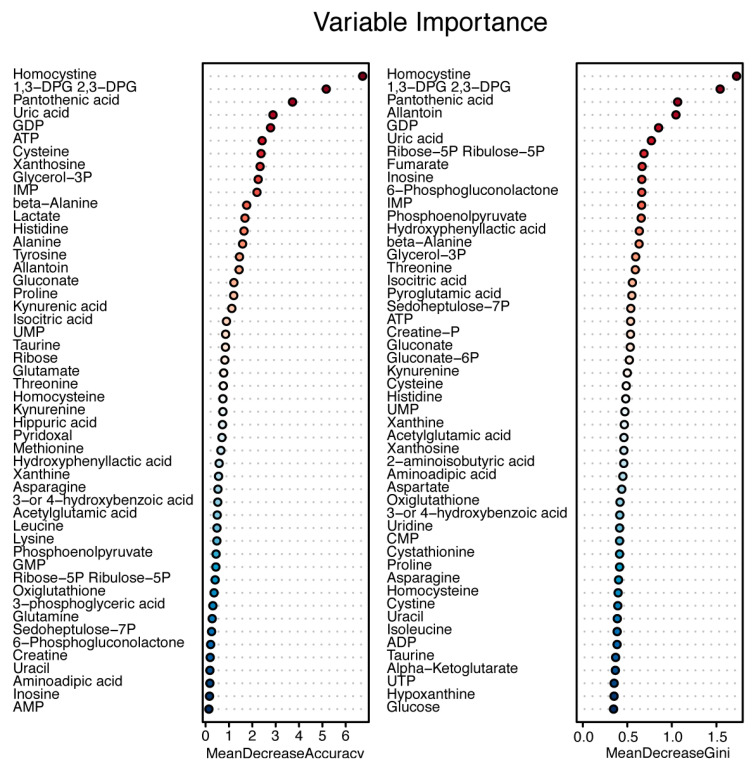
Variable importance plot from random forest analysis. Variable importance was determined using the mean decrease in accuracy and mean decrease in Gini score upon removal of the variable.

Additionally, non-linear relationships between metabolites and response were tested using a random forest analysis. Variable importance was determined according to the decrease in accuracy and Gini score upon removal of variables from the models tested. The most significant variables again were homocystine and 1,3-DPG/2,3-DPG (Figure 4).

### 3.4. Enrichment Analysis

**Figure 5:**
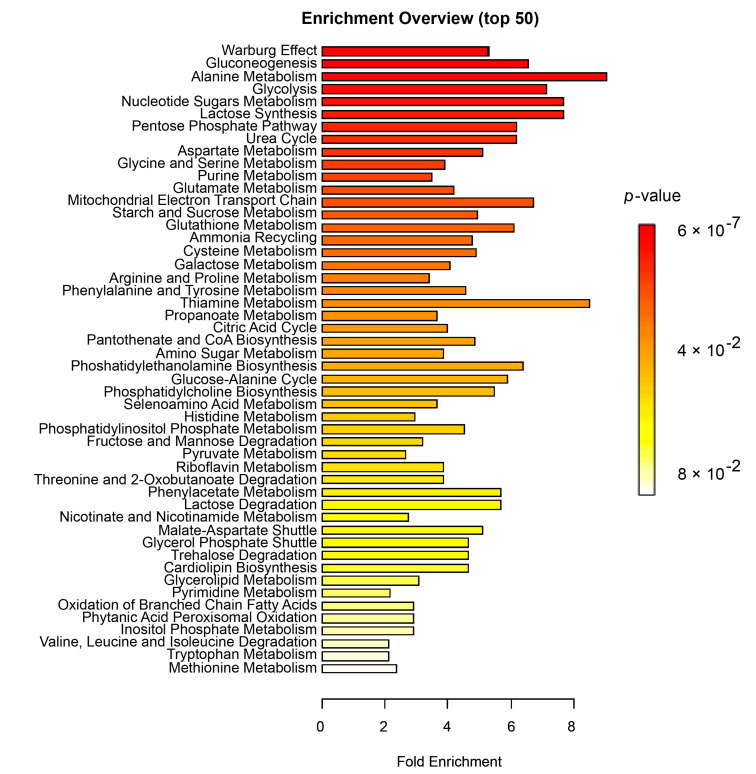
Results of overrepresentation analysis (ORA) between insufficient and sufficient responders to MTX. Summary of overrepresentation analysis results at baseline in relation to response to MTX at 3 months. The *X*-axis shows the fold enrichment between response groups and the color indicates the significance level, where red is most significant. *p*-values < 0.05 were considered significant. For details on the number of metabolites per pathway, see Supplementary Table S2.

Finally, to examine whether certain cellular processes were overrepresented in insufficient versus sufficient responders, we performed an overrepresentation analysis (ORA). Metabolites with a VIP score >1 (Table S1) were included in the analysis. The most significantly enriched metabolic events were related to cellular respiration: Warburg effect (FDR_padjust_ = 5.59 × 10^−5^), gluconeogenesis (FDR_padjust_ = 1.38 × 10^−4^), glycolysis (FDR_padjust_ = 5.69 × 10^−4^), lactose synthesis (FDR_padjust_ = 8.22 × 10^−4^), pentose phosphate pathway (FDR_padjust_ = 8.22 × 10^−4^), urea cycle (FDR_padjust_ = 8.22 × 10^−4^) and to amino acid metabolism (Figure 5 and Supplementary Table S2).

## 4. Discussion

In this study, we examined metabolite profiles prior to treatment initiation in early RA patients to identify potential biomarkers for response to MTX. At baseline, significantly different concentrations were observed between insufficient responders and sufficient responders in eight metabolites. Homocystine, taurine, ATP, GDP and uric acid concentrations were significantly lower in insufficient responders, while glycolytic intermediates 1,3-DPG/2,3-DPG, glycerol-3-phosphate and phosphoenolpyruvate (PEP) were significantly higher in sufficient responders. The most promising biomarkers, homocystine, glycerol-3-phosphate and 1,3-DPG/2,3-DPG, together constructed a ROC with high AUC of 0.81 (95% CI: 0.72–0.91) and sensitivity of 78% and specificity of 76%. Furthermore, overrepresentation analysis indicated that metabolic processes related to cellular respiration and amino acid metabolism at baseline were potentially associated with treatment response, which might be interesting pathways to further explore in MTX-based therapies for RA.

In this study, lower baseline plasma levels of uric acid and taurine were related to insufficient response to MTX. Uric acid concentrations should be interpreted with caution in this study, as the analytical variation for this metabolite exceeded 25%. Uric acid was also previously quantified in 226 patients receiving MTX in the tREACH dataset, measured using a routine chemistry method on a Roche Cobas 8000 system (Roche, Almere, Netherlands) [10]. In this set, uric acid was borderline insignificant in a crude logistic regression model (OR = 0.04, 95% CI: 0.00–1.66, *p* = 0.09) and when adjusted for baseline DAS28 (OR = 0.02, 95% CI: 0.00–1.16, *p* = 0.06). Although not significant, the effect sizes pointed in the same direction as findings in the current study, suggesting that uric acid might play a role in response to MTX. This result is also in agreement with a study by Wang et al., who assessed 38 early RA patients on MTX monotherapy (13 insufficient responders versus 25 sufficient responders) at baseline and at 24 weeks [31].

The same trend was observed for taurine in the present study and the one by Wang et al. [31]. Interestingly, for taurine, the opposite was observed in serum samples of established RA patients, where taurine levels were lower in sufficient responders prior to TNFα inhibitor initiation after insufficient response to DMARD therapy [20]. Although these studies support taurine as a potential biomarker to choose between therapies, it has to be considered that the latter study was performed in a group of established RA patients from whom it was not clear what the effect of previous DMARD use was on the metabolite concentrations. In the same study [20], glycerol-3-phosphate was lower in sufficient responders at the start of TNFα inhibitor initiation, which is consistent with our findings that glycerol-3-phosphate was higher in insufficient responders at the start of MTX combination therapy, suggesting that insufficient responders to MTX with low glycerol-3-phosphate may be insufficient responders to TNFα inhibitors as well. Sasaki and colleagues [19] also observed higher glycerol-3-phosphate levels in the plasma of RA patients versus non RA controls; however, they did not observe differences in relation to response to MTX and/or corticosteroid therapy. This may be due to the small group sizes of patients receiving MTX (*n* = 27 sufficient responders versus n = 12 insufficient responders). Plasma amino acid metabolites that were previously described in relation to DAS28 by Smolenska et al. [17], such as threonine, tryptophan (positive correlation) and histidine and phenylalanine (negative correlation), could also separate insufficient and sufficient responders in our study (Figure 4). However, we did note that the Gini score was largely unaltered upon removal of threonine, tryptophan, histidine and phenylalanine compared to other metabolites in the variable importance plots (e.g., homocystine and 1,3-DPG; Figure 4). This means that threonine, tryptophan, histidine and phenylalanine were less important in discriminating insufficient responders compared to metabolites ranked higher in the variable importance plots. However, the intercorrelation between metabolites can influence their contribution to the model and their ranking in Figure 4. This may, for instance, apply to taurine, which seems to have only minor importance in the random forest analysis but was significantly different between response groups at baseline (0.021) and had a VIP score of (1.607; Table S1). However, taurine is highly correlated to GDP and ATP (Figure 2); hence, the inclusion of GDP and ATP in the model in the random forest analysis made taurine redundant in this case (Figure 4).

From the most successful, 1,3-DPG/2,3-DPG has not been previously described in relation to response to MTX treatment. Homocystine consists of two homocysteine molecules connected by a disulfide bond [32]. Previous studies showed that homocysteine concentrations increase upon MTX treatment in RA, while concentrations are reduced again by supplementation with folic acid [33,34], which is prescribed to RA patients to avoid adverse events. Total homocysteine is quantified as a mixture of all bound and unbound homocysteine molecules, including homocystine, which is first reduced to free homocysteine components. Higher total homocysteine levels could therefore be influenced by higher homocystine levels. Total homocysteine was also previously quantified in the plasma samples of 285 individuals from the tREACH study [23]; however, no relation was observed between homocysteine and response to MTX. Moreover, homocystine from the current metabolomic study and previously observed total homocysteine levels in the same individuals did not correlate (R = 0.03, *p* = 0.77). The precise role of homocystine in relation to response to MTX warrants further investigation.

Under normal physiological circumstances, phosphorylated metabolites are usually maintained intracellularly. There could be several reasons that phosphorylated metabolites were identified in plasma samples analyzed in this study. Inflammatory/oxidative stress conditions related to the pathogenesis of RA have been reported to trigger the extracellular release of lactate, ATP, ADP and AMP [14]. These extracellular adenine nucleotides represent a potential pro-inflammatory metabolite during the early stages of RA [35]. However, ectophosphatases CD73 and CD39 on immune-competent cells, or alkaline phosphatase, can convert extracellular ATP, ADP and AMP into adenosine, which acts as an anti-inflammatory regulator via interaction with adenosine receptors on leukocytes [36,37]. Accordingly, low CD39 expression on regulatory T-cells has been identified as a biomarker for MTX resistance in RA [38,39].

Furthermore, parallel changes in glycerol-3-phosphate, 2,3-DPG and PEP in good and poor responders point to alterations in glycolysis at the level of the regulatory enzyme pyruvate kinase (PK). In fact, RBC enzymopathies due to PK deficiency are characterized by increased levels of glycerol-3-phosphate, 2,3-DGP and PEP (and low ATP/GTP) [40,41] whereas enzymopathies due to hyperactive PK activity feature marked downregulation of the three glycolytic intermediates (but high ATP/GTP) [42].

To better understand the biological relevance of our findings, an overrepresentation analysis was performed, of which the results should be considered as exploratory given that solely metabolites with VIP > 1 were included and not all metabolites were significantly different at baseline. From this perspective, results from the overrepresentation analysis showed that differences in baseline metabolites in relation to MTX response were primarily involved in the Warburg effect and glycolysis. These findings are consistent with recent studies in the field of “immunometabolism”, describing alternate metabolic signatures during the activation of immune cells and autoimmune pathogenesis [15,43]. Especially the Warburg effect, describing a shift towards inefficient energy production through aerobic glycolysis, and well recognized for its impact on drug response in cancer cells [44], has been extensively described in RA patients, as well as the upregulation of glycolysis [45,46,47,48,49]. As these processes have been associated with a proinflammatory state, targeting the Warburg effect or glycolysis has been suggested as a potential RA therapy [50,51,52,53]. However, these processes have, to date, not been linked to the response to existing therapies in RA. The results of our study suggest that there may be a subgroup within early RA patients prior to treatment in which the Warburg effect and enhanced glycolysis could play a role in relation to response to MTX combination therapy. Moreover, MTX is a metabolite inhibitor itself, with primary targets in the folate/one-carbon metabolism pathway (e.g., dihydrofolate reductase (DHFR), thymidylate synthase (TYMS) and 5-aminoimidazole-4-carboxamide ribonucleotide formyltransferase/IMP cyclohydrolase (ATIC)), which have many downstream effects, varying per immune cell type. For instance, as reviewed by Cronstein and Aune [5], MTX indirectly inhibits NF-κB activity in T-cells through the induction of long intergenic non-coding RNA p21 (lncRNA-p21). Interestingly, lncRNA-p21 also promotes HIF1-α upregulation under hypoxic circumstances, which regulates the Warburg effect [54]. This might be an interesting link between response to MTX and the Warburg effect that deserves further investigation.

Strengths of this study were that it consisted of two equal groups with extremes in responses to MTX, which allowed us to identify the largest differences between response groups. Secondly, we used an untargeted approach, which led to new insights into possible metabolic biomarkers and pathways involved in the response to MTX. Furthermore, the study was performed on blood plasma samples, which are easily accessible for routine biomarker purposes. Limitations to this study were that it was performed using a semi-quantitative assay, meaning that metabolite concentrations cannot be directly compared with measurements by other methods and in other studies, but only between response groups in the same study. Moreover, our sample size was limited; thus, validation using other methods is warranted. Finally, correlations between top findings and BMI were low (Pearson’s r < 0.3), and due to the low number of patients per group, we did not take into consideration other factors such as comorbidity, food intake and lifestyle factors, such as smoking, which may have influenced metabolic profiles [55].

For future studies, it would also be interesting to examine metabolite samples longitudinally. As a predictor for response, baseline samples are most suitable, as treatment adjustments can be made from the start of treatment initiation when appropriate. However, to obtain a better understanding of MTX’s mechanism(s) of action and, in particular, its effect on metabolic processes, it would be interesting to follow metabolites longitudinally before and after MTX in relation to treatment response. This approach may reveal certain biomarkers that could possibly also serve as early markers for response during the first few months of treatment. A decrease in uric acid, for instance, has been observed in good responders to MTX in RA patients [56]. This, together with our results demonstrating that lower uric acid levels in insufficient responders were observed at baseline, could indicate that MTX acts better when certain pathways are upregulated prior to treatment. Nevertheless, both results first require validation.

Up to now, many other baseline variables have been assessed in relation to MTX response without much success, as previously reviewed [5,9,57,58]. Ideally, biomarkers should be combined in prediction models, including clinical, laboratory and lifestyle parameters [11,59,60]. Conceivably, metabolomic biomarkers for MTX response could be used as standalone or in addition to such a prediction model to identify insufficient responders prior to treatment and enabling prescription of step-up treatment from the start.

## Figures and Tables

**Table 1:** Characteristics of rheumatoid arthritis patients with insufficient response versus sufficient response to MTX (combination) therapy.

	Insufficient Responders (DAS28-ESR > 3.2) N = 41	Sufficient Responders (DAS28-ESR ≤ 3.2) N = 41	*p*-Value
Baseline DAS28, mean ± SD	4.3 ± 1.3	5.6 ± 1.0	<0.001
Age, mean ± SD	50.0 ± 11.9	52.6 ± 16.9	0.41
Sex, Male, N (%)	8 (20)	15 (37)	0.14
BMI (kg/m^2^), mean ± SD	28.1 ± 5.4	24.3 ± 4.1 ^#^	<0.001
RF positive, N (%)	26 (63)	33 (80)	0.14
ACPA positive, N (%)	25 (61)	31 (76)	0.24
Treatment			
MTX + SSZ + HCQ + corticosteroids i.m.	8 (20)	15 (37)	0.14
MTX + SSZ + HCQ + corticosteroids per os	11 (27)	15 (37)	0.48
MTX + corticosteroids per os	13 (32)	7 (17)	0.20
MTX	9 (22)	4 (10)	0.23

^#^ BMI, N = 1 missing value, BMI = body mass index, MTX = methotrexate, SSZ = sulfasalazine, HCQ = hydroxychloroquine, i.m. = intramuscular. RF = rheumatoid factor, ACPA = anti-citrullinated protein antibody

## Supplementary Materials

The following are available online at https://www.mdpi.com/2075-4426/10/4/271/s1, Table S1: List of most important metabolites in discriminating insufficient responders from sufficient responders, Figure S1: PLS-DA analysis of baseline metabolites and insufficient and sufficient responders to MTX, determined at 3 months, **Table S2**: Results of overrepresentation analysis at baseline in relation to response to MTX at 3 months in early RA patients.
