# Supplementary material for: Identification of Metabolic Biomarkers in Relation to Methotrexate Response in Early Rheumatoid Arthritis
Source: J Pers Med. 2020 Dec 10;10(4):271. doi: 10.3390/jpm10040271 (PMC7768454; doi:10.3390/jpm10040271)
Supplement: Supplementary file 1 [file jpm-10-00271-s001.pdf]

Supplementary Files

Table S1. List of most important metabolites in discriminating insufficient responders from sufficient responders.

Metabolite	HMDB id	P-value	FDR	VIP score	Log2 fold change
Increased in insufficient responders					
1,3-Diphosphoglyceric acid (1,3-DPG)	HMDB0001270	0.001	0.100	2.439	0.398
2,3-Diphosphoglyceric acid (2,3-DPG)	HMDB0001294				
Glycerol-3-phosphate	HMDB0000126	0.039	0.439	1.605	0.264
Phosphoenolpyruvate (PEP)	HMDB0000263	0.048	0.439	1.503	0.162
6-Phosphogluconolactone	HMDB0001127	0.181	0.567	1.503	0.254
Phosphocreatine	HMDB0001511	0.497	0.781	1.462	0.103
Kynurenine	HMDB0000684	0.841	0.890	1.377	0.023
Ribose-5-phosphate and	HMDB0001548	0.063	0.439	1.354	0.252
Ribulose-5-phosphate and	HMDB0000618				
Xylulose-5-phosphate	HMDB0006212				
Inosine monophosphate (IMP)	HMDB0000175	0.054	0.439	1.344	0.199
Threonine	HMDB0000167	0.102	0.506	1.306	0.117
Histidine	HMDB0000177	0.072	0.439	1.295	0.210
Oxiglutathione	HMDB0003337	0.395	0.781	1.268	0.231
Allantoin	HMDB0000462	0.114	0.511	1.109	0.596
Sarcosine	HMDB0000271	0.410	0.781	1.068	0.068
Alanine	HMDB0000161	0.410	0.781	1.067	0.068
Hydroxyphenyllactic acid	HMDB0000755	0.169	0.567	1.036	0.504
Alpha-Ketoglutarate	HMDB0000208	0.287	0.730	1.035	0.347
Decreased in insufficient responders					
Homocystine	HMDB0000575	0.004	0.200	1.927	-0.149
Taurine	HMDB0000251	0.021	0.439	1.607	-0.219
Adenosine triphosphate (ATP)	HMDB0000538	0.046	0.439	1.552	-0.898
Guanosine diphosphate (GDP)	HMDB0001201	0.040	0.439	1.540	-1.647
ADP	HMDB0001341	0.066	0.439	1.484	-1.214
Uric acid	HMDB0000289	0.030	0.439	1.455	-0.249
Guanosine monophosphate (GMP)	HMDB0001397	0.116	0.511	1.453	-0.612
Cytidine monophosphate (CMP)	HMDB0000095	0.053	0.439	1.441	-0.305
Adenosine monophosphate (AMP)	HMDB0000045	0.075	0.439	1.393	-0.678
Uridine triphosphate (UTP)	HMDB0000285	0.329	0.755	1.361	-0.093
Ribose	HMDB0000283	0.057	0.439	1.311	-0.214
Glucose-6-phosphate	HMDB0001401	0.246	0.660	1.293	-0.108
Cystathionine	HMDB0033142	0.067	0.439	1.251	-0.441
Fumarate	HMDB0000134	0.079	0.439	1.249	-0.144
Glutamate	HMDB0000148	0.120	0.511	1.207	-0.406
Hexose-phosphate	HMDB0000645	0.498	0.781	1.146	-0.094
	HMDB0001078				
	HMDB0001586				
	HMDB0006330				
2-phosphoglyceric acid	HMDB0000362	0.303	0.748	1.134	-0.225
Valine	HMDB0000883	0.162	0.567	1.119	-0.114
Uridine 5'-monophosphate (UMP)	HMDB0000288	0.102	0.506	1.103	-0.220
Sedoheptulose-7-phosphate	HMDB0001068	0.750	0.890	1.027	-0.118

Presented metabolites with VIP score >1 generated from the PLS-DA analysis. P-values are the result of Welch's t-test per metabolite between good and poor responders prior to MTX treatment.

Figure S1 PLS-DA analysis of baseline metabolites and insufficient and sufficient responders to MTX, determined at 3 months

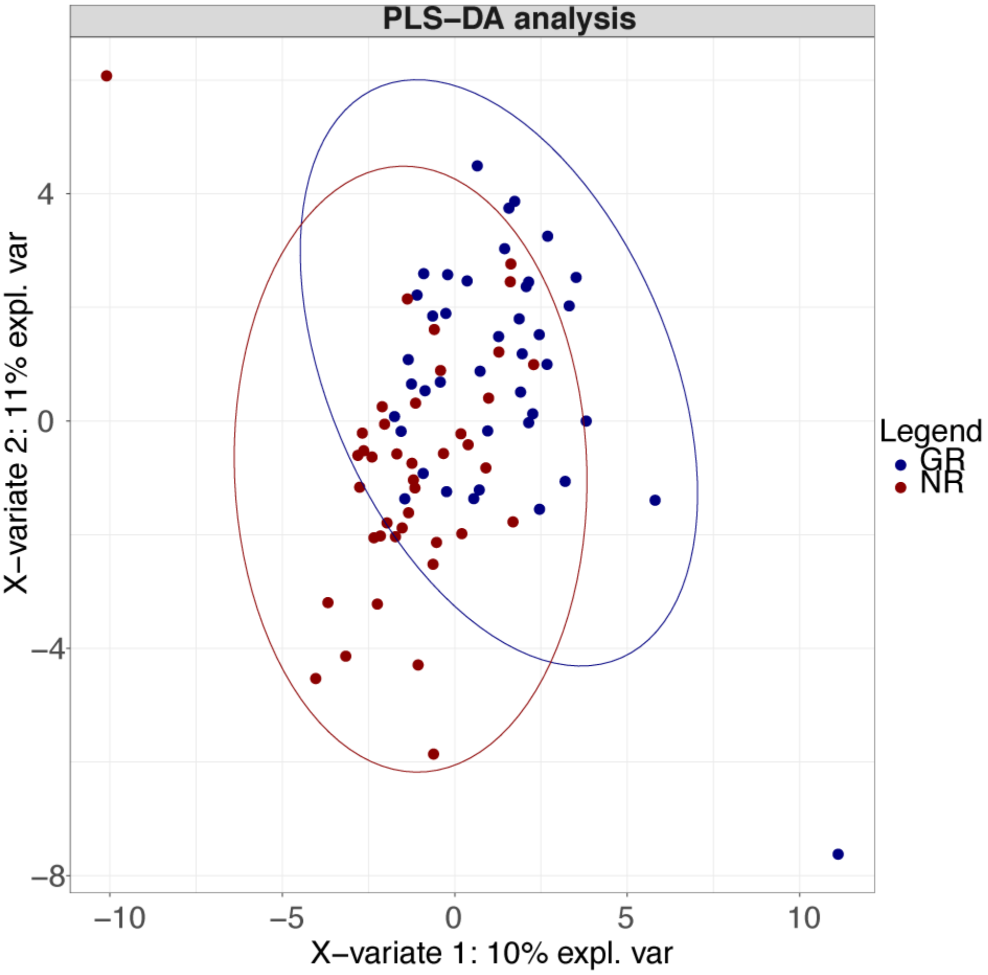

Table S2. Results of Over Representation Analysis at baseline in relation to response to MTX at 3 months in early RA patients.

Metabolic event	Total	Exp.	Hits	Raw p	Holm p	FDR
Warburg Effect	58	2.27	12	5.71E-07	5.59E-05	5.59E-05
Gluconeogenesis	35	1.37	9	2.82E-06	2.74E-04	1.38E-04
Alanine Metabolism	17	0.66	6	2.19E-05	2.10E-03	5.69E-04
Glycolysis	25	0.98	7	2.32E-05	2.21E-03	5.69E-04
Nucleotide Sugars Metabolism	20	0.78	6	6.27E-05	5.90E-03	8.22E-04
Lactose Synthesis	20	0.78	6	6.27E-05	5.90E-03	8.22E-04
Pentose Phosphate Pathway	29	1.13	7	6.71E-05	6.17E-03	8.22E-04
Urea Cycle	29	1.13	7	6.71E-05	6.17E-03	8.22E-04
Aspartate Metabolism	35	1.37	7	2.43E-04	2.19E-02	2.49E-03
Glycine and Serine Metabolism	59	2.30	9	2.54E-04	2.26E-02	2.49E-03
Purine Metabolism	74	2.89	10	3.07E-04	2.70E-02	2.73E-03
Glutamate Metabolism	49	1.91	8	3.67E-04	3.19E-02	3.00E-03
Mitochondrial Electron Transport Chain	19	0.74	5	5.48E-04	4.72E-02	4.13E-03
Starch and Sucrose Metabolism	31	1.21	6	8.65E-04	7.35E-02	5.92E-03
Glutathione Metabolism	21	0.82	5	9.06E-04	7.61E-02	5.92E-03
Ammonia Recycling	32	1.25	6	1.03E-03	8.58E-02	6.33E-03
Cysteine Metabolism	26	1.02	5	2.53E-03	2.08E-01	1.44E-02
Galactose Metabolism	38	1.48	6	2.64E-03	2.14E-01	1.44E-02
Arginine and Proline Metabolism	53	2.07	7	3.29E-03	2.63E-01	1.70E-02
Phenylalanine and Tyrosine Metabolism	28	1.09	5	3.57E-03	2.82E-01	1.75E-02
Thiamine Metabolism	9	0.35	3	3.94E-03	3.08E-01	1.84E-02
Propanoate Metabolism	42	1.64	6	4.47E-03	3.44E-01	1.99E-02
Citric Acid Cycle	32	1.25	5	6.52E-03	4.95E-01	2.78E-02
Pantothenate and CoA Biosynthesis	21	0.82	4	7.39E-03	5.55E-01	2.92E-02
Amino Sugar Metabolism	33	1.29	5	7.46E-03	5.55E-01	2.92E-02
Phosphatidylethanolamine Biosynthesis	12	0.47	3	9.52E-03	6.95E-01	3.59E-02
Glucose-Alanine Cycle	13	0.51	3	1.20E-02	8.67E-01	4.37E-02
Phosphatidylcholine Biosynthesis	14	0.55	3	1.49E-02	1.00E+00	5.22E-02
Selenoamino Acid Metabolism	28	1.09	4	2.07E-02	1.00E+00	7.01E-02
Histidine Metabolism	43	1.68	5	2.27E-02	1.00E+00	7.40E-02
Phosphatidylinositol Phosphate Metabolism	17	0.66	3	2.57E-02	1.00E+00	8.12E-02
Fructose and Mannose Degradation	32	1.25	4	3.25E-02	1.00E+00	9.96E-02
Pyruvate Metabolism	48	1.88	5	3.49E-02	1.00E+00	1.04E-01
Riboflavin Metabolism	20	0.78	3	3.97E-02	1.00E+00	1.11E-01
Threonine and 2-Oxobutanoate Degradation	20	0.78	3	3.97E-02	1.00E+00	1.11E-01
Phenylacetate Metabolism	9	0.35	2	4.50E-02	1.00E+00	1.19E-01
Lactose Degradation	9	0.35	2	4.50E-02	1.00E+00	1.19E-01
Nicotinate and Nicotinamide Metabolism	37	1.45	4	5.19E-02	1.00E+00	1.34E-01
Malate-Aspartate Shuttle	10	0.39	2	5.49E-02	1.00E+00	1.38E-01
Glycerol Phosphate Shuttle	11	0.43	2	6.55E-02	1.00E+00	1.53E-01
Trehalose Degradation	11	0.43	2	6.55E-02	1.00E+00	1.53E-01
Cardiolipin Biosynthesis	11	0.43	2	6.55E-02	1.00E+00	1.53E-01
Glycerolipid Metabolism	25	0.98	3	7.00E-02	1.00E+00	1.58E-01

Pyrimidine Metabolism	59	2.30	5	7.44E-02	1.00E+00	1.58E-01
Oxidation of Branched Chain Fatty Acids	26	1.02	3	7.70E-02	1.00E+00	1.58E-01
Phytanic Acid Peroxisomal Oxidation	26	1.02	3	7.70E-02	1.00E+00	1.58E-01
Inositol Phosphate Metabolism	26	1.02	3	7.70E-02	1.00E+00	1.58E-01
Valine, Leucine and Isoleucine Degradation	60	2.34	5	7.88E-02	1.00E+00	1.58E-01
Tryptophan Metabolism	60	2.34	5	7.88E-02	1.00E+00	1.58E-01
Methionine Metabolism	43	1.68	4	8.20E-02	1.00E+00	1.61E-01
Folate Metabolism	29	1.13	3	9.99E-02	1.00E+00	1.92E-01
Inositol Metabolism	33	1.29	3	1.34E-01	1.00E+00	2.53E-01
Beta Oxidation of Very Long Chain Fatty Acids	17	0.66	2	1.40E-01	1.00E+00	2.59E-01
Beta-Alanine Metabolism	34	1.33	3	1.43E-01	1.00E+00	2.60E-01
Methylhistidine Metabolism	4	0.16	1	1.48E-01	1.00E+00	2.63E-01
Butyrate Metabolism	19	0.74	2	1.68E-01	1.00E+00	2.88E-01
Ethanol Degradation	19	0.74	2	1.68E-01	1.00E+00	2.88E-01
Sulfate/Sulfite Metabolism	22	0.86	2	2.11E-01	1.00E+00	3.50E-01
Transfer of Acetyl Groups into Mitochondria	22	0.86	2	2.11E-01	1.00E+00	3.50E-01
Biotin Metabolism	8	0.31	1	2.74E-01	1.00E+00	4.47E-01
Mitochondrial Beta-Oxidation of Short Chain Saturated Fatty Acids	27	1.05	2	2.85E-01	1.00E+00	4.50E-01
Mitochondrial Beta-Oxidation of Medium Chain Saturated Fatty Acids	27	1.05	2	2.85E-01	1.00E+00	4.50E-01
Mitochondrial Beta-Oxidation of Long Chain Saturated Fatty Acids	28	1.09	2	3.00E-01	1.00E+00	4.56E-01
Homocysteine Degradation	9	0.35	1	3.02E-01	1.00E+00	4.56E-01
De Novo Triacylglycerol Biosynthesis	9	0.35	1	3.02E-01	1.00E+00	4.56E-01
Lysine Degradation	30	1.17	2	3.29E-01	1.00E+00	4.89E-01
Taurine and Hypotaurine Metabolism	12	0.47	1	3.82E-01	1.00E+00	5.58E-01
Sphingolipid Metabolism	40	1.56	2	4.70E-01	1.00E+00	6.72E-01
Bile Acid Biosynthesis	65	2.54	3	4.73E-01	1.00E+00	6.72E-01
Fatty acid Metabolism	43	1.68	2	5.09E-01	1.00E+00	7.11E-01
Spermidine and Spermine Biosynthesis	18	0.70	1	5.15E-01	1.00E+00	7.11E-01
Tyrosine Metabolism	72	2.81	3	5.44E-01	1.00E+00	7.41E-01
Steroid Biosynthesis	48	1.88	2	5.70E-01	1.00E+00	7.56E-01
Betaine Metabolism	21	0.82	1	5.71E-01	1.00E+00	7.56E-01
Carnitine Synthesis	22	0.86	1	5.88E-01	1.00E+00	7.68E-01
Plasmalogen Synthesis	26	1.02	1	6.50E-01	1.00E+00	8.38E-01
Phospholipid Biosynthesis	29	1.13	1	6.90E-01	1.00E+00	8.78E-01
Arachidonic Acid Metabolism	69	2.70	2	7.67E-01	1.00E+00	9.64E-01

Compound names of metabolites with VIP score >1 were used as input. The small molecule pathway database was selected as reference library. Hits= number of metabolites that were enriched in the pathway. Expected is number of hits expected in the pathway due to chance. FDR=False Discovery Rate. FDR- adjusted p-values <0.05 were considered significant.
